# Aerial medical evacuation of health workers with suspected Ebola virus disease in Guinea Conakry-interest of a negative pressure isolation pod-a case series

**DOI:** 10.1186/s12873-017-0121-x

**Published:** 2017-03-11

**Authors:** Jean-Michel Dindart, Olivier Peyrouset, Romain Palich, Abdoul Bing, Richard Kojan, Solenne Barbe, Souley Harouna, Nikki Blackwell

**Affiliations:** 1 Alliance for International Medical Action, Dakar, Senegal; 2 CHU de Bordeaux, University of Bordeaux, Talence, France; 3 University of Queensland, Brisbane, QLD Australia

**Keywords:** Air transit isolator, Ebola, Guinea, Transportation of patients, Haemorrhagic fever, Infection control

## Abstract

We report 4 cases of Health Workers (HW) suspected of having contracted Ebola Virus Disease (EVD), transported from the Alliance for International Medical Action (ALIMA) Ebola Treatment Centre (ETC) in N’Zerekore, Guinea to the Treatment Centre for Carers run by the medical corps of the French army in Conakry, the capital of Guinea, which was established on 17 January 2015 and closed on 7 July 2015. In total more than 500 HWs have died from EVD since the epidemic began. This mortality has had significant effects on the ability of local services to respond appropriately to the disaster. The HWs were transported by air in the “Human Stretcher Transit Isolator-Total Containment (Oxford) Limited” (HSTI-TCOL) negative pressure isolation pod. Medical evacuation of patients with suspected, potentially fatal, infectious diseases is feasible with the use of a light isolator for patients without critical dysfunctions.

## Background

West Africa has been ravaged by an epidemic of Ebola virus disease (EVD) since March 2014. In Guinea, 3700 HWs have been involved in the fight against Ebola. Viral transmission occurs by direct contact with infected body fluids. In an EVD outbreak, the key is to isolate and manage affected patients as quickly as possible in an ETC. In West Africa, 881 HWs had been infected, of whom 513 (58%) had died [1]. The cumulative incidence of EVD is 21–32 times greater for HWs than the general population [2]. We report 4 cases of HWs suspected of EVD, transported by air, the only practical means of transport, from the ETC in N’Zerekore, Guinea, to the Treatment Centre for Carers (CTS—Centre de Traitement de Soignants) run by the French army medical corps in Conakry, with critical care facilities. The HWs were transported by air in the “Human Stretcher Transit Isolator-Total Containment (Oxford) Limited” (HSTI-TCOL) negative pressure isolation pod, manufactured by TCOL company. Road transport was considered unsuitable because of the time required (2 days) and security risks.

**Case Number 1**: a nurse working in the ETC had contact with numerous patients with EVD in the “high risk zone” of the ETC without any reported biosafety incident. She developed profuse diarrhoea, and fever of 38.5C. Given this clinical presentation which is seen in early EVD [3–5], and the history of contact with patients infected with Ebola virus, she was managed in isolation. Examination was unremarkable, without signs of distress. A peripheral intravenous (IV) cannula was placed, blood specimens for malaria rapid test and a RT-PCR for Ebola (reverse transcription polymerase chain reaction, Altona Diagnostics) were sent to the adjacent laboratory run by the Belgian Organisation B-FAST (Belgian First Aid and Support). With her consent, it was decided to evacuate her to the CTS before the results of the RT-PCR [6].

She was transported to N’Zerekore airport by a dedicated ambulance. She was assisted in entering the isolation pod by two doctors, in full personal protective equipment (PPE), after the arrival of the evacuation helicopter. The United Nations (UN) helicopter provided by the World Food Program, which was the only aircraft available arrived at 12:30 AM and took off at 01:30 PM. In flight, the patient’s temperature rose to 40C. On arrival at the CTS at 5:30 PM, her care was taken over by the CTS team. Blood results were available on landing in Conakry; the first PCR for Ebola was negative as was the rapid test for malaria. PCR 48 h later was also negative. She was discharged after 3 days with a diagnosis of severe diarrhea, treated with ciprofloxacin.

## Case number 2-3-4

These 3 HWs, working in the ETC had no medical history, and had close contacts, in full PPE, with numerous patients with EVD. In “the high risk zone”, no biosafety incident reported.

- **Case No. 2**, a doctor, noted a fever of 38.5C with a lumbar and low thoracic pain. She was very nauseous and her temperature rose to 38.9C in flight. The rapid test of malaria (HRP2), the first RT-PCR and subsequently 48 h were negative. The diagnostic was right lower lobe pneumonia. She left the CTS on oral amoxicillin/clavulanic acid therapy after 48 h of IV ceftriaxone, and oral ciprofloxacin.
- **Case No. 3**, a nurse, presented acute severe profuse liquid diarrhoea, nausea. She vomited six times, had abdominal pain and a temperature of 37.4C. For a fortnight she had had close contact, wearing full PPE, with an EVD patient who had diarrhoea and haemorrhage. Very nauseated and anxious in flight, her temperature was 37.6C. The rapid diagnostic test for malaria and initial RT-PCR thus 48 h were negative. She left the CTS after a 72 h symptomatic treatment.
- **Case No. 4**, worked as a hygienist in the ETC. He presented fever to 39C, severe listlessness, profuse sweating, vomiting and abdominal pain. He had worked in full PPE, including handling contaminated wastes for destruction in the incinerator. He did not take malaria prophylaxis but slept under a mosquito net. In flight, we noticed asthenia and drowsiness [Table 1]. Tests performed in N’Zerekore revealed a negative RT—PCR for Ebola with positive rapid test of malaria (HRP2). After a second negative test RT-PCR at 48 h, he was discharged from the CTS with anti-malarial treatment.

**Table 1:** Patient and isolation pod parameters during transfer (Patient 4)

Time	17.15	17.20	17.40	18.25	18.50	19.25
Patient temperature (C)	Take-off		37.2	37.5	36.9	Landing
Respiratory rate per minute		34	26	29	22	
Level of consciousness		Drowsy	Alert	Alert	Alert	
Glasgow Coma Scale		15	15	15	15	
Internal temperature of the isolation pod (C)		35.8	32	26.6	22	
Negative pressure of isolation pod (mmH2O)		4	4	3.5	4	
Hygrometry inside the isolation pod			41
  Support for cases 2, 3 and 4 was identical : isolation and peripheral access with cristalloid infusion, symptomatic oral or IV treatment; blood was taken for biochemistry, rapid test for malaria (HRP2), RT—PCR for Ebola, and blood electrolytes (I-Stat Chem 8 + cartridges, Abbott Point of Care® Inc.).

## Evacuation protocol

**Fig. 1:**
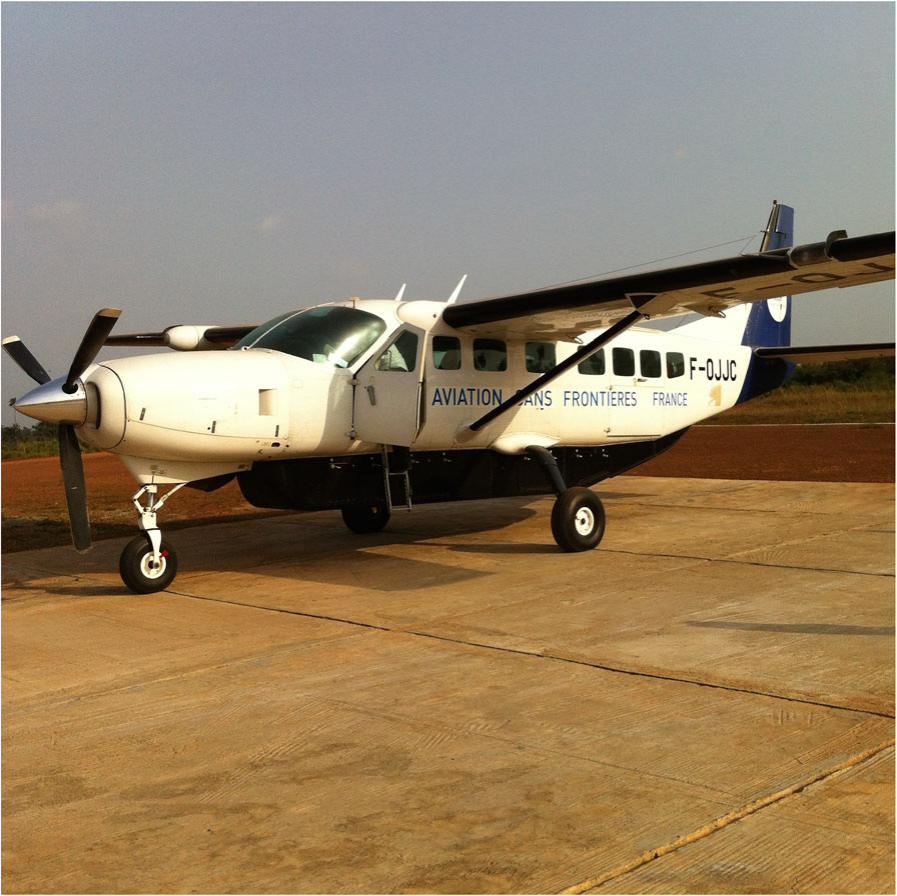
Cessna 208 Caravan F-OJCC used for the evacuation of patients 2, 3 and 4

**Fig. 2:**
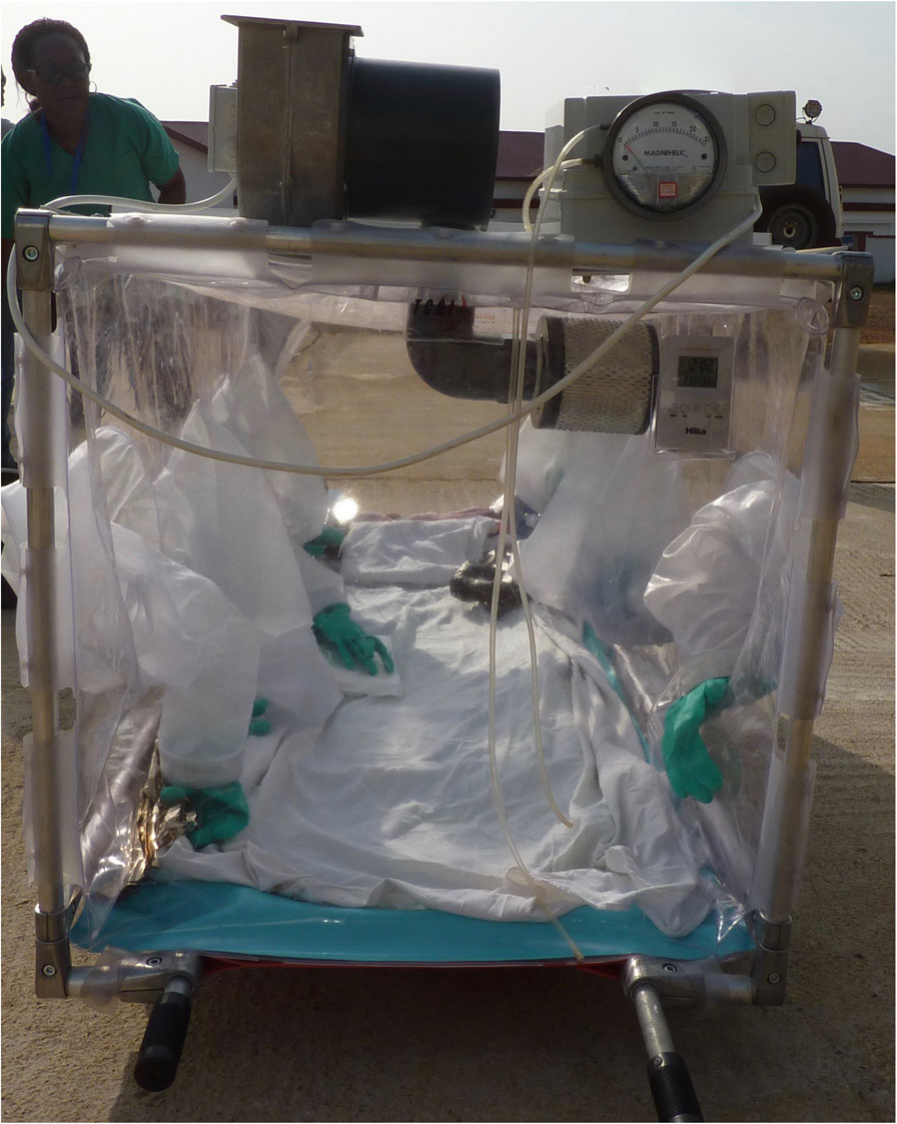
The isolation pod HSTI-TCOL is prepared on the airstrip close to the aircraft

**Fig. 3:**
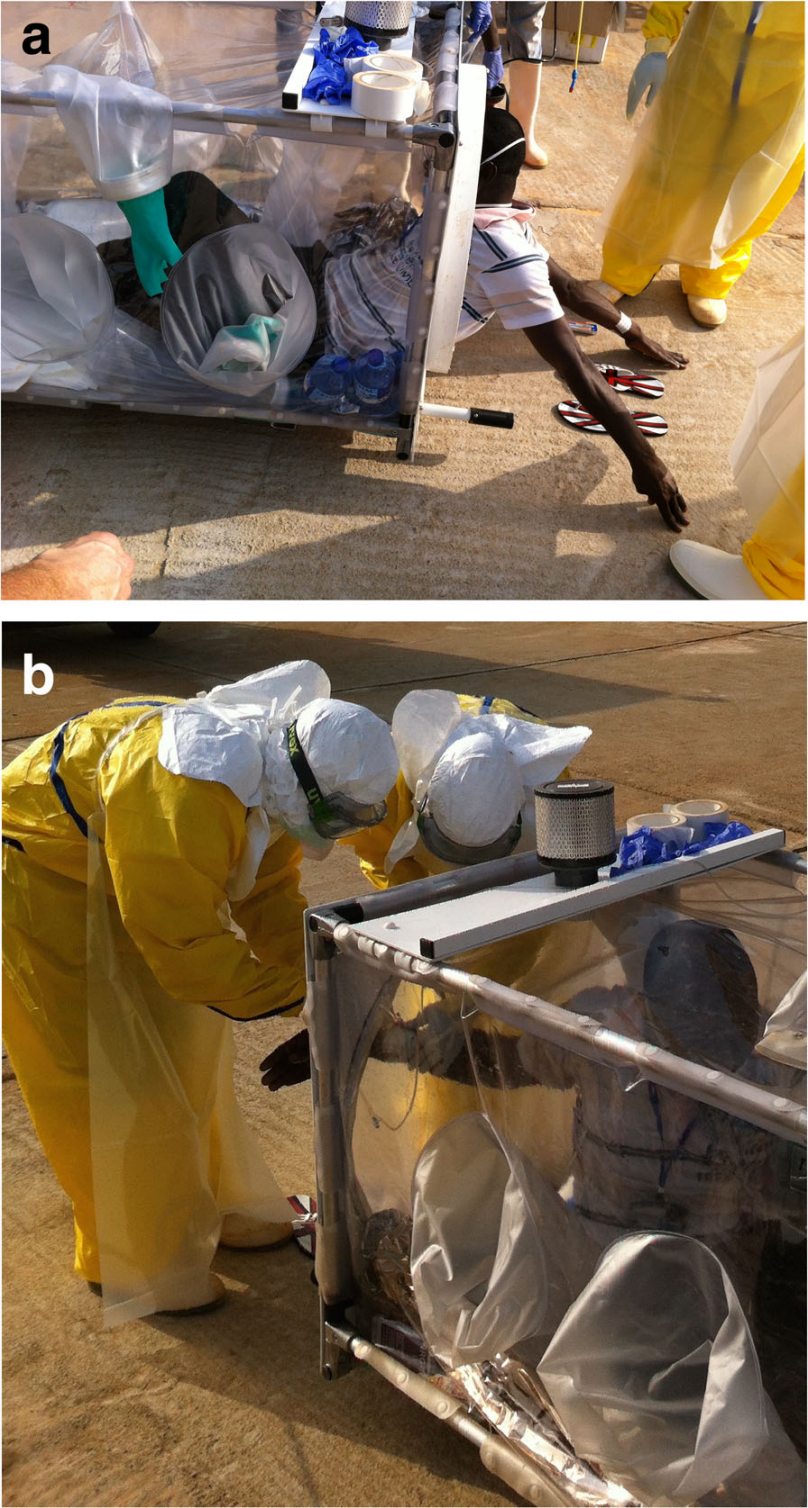
**a & b** The patient is loaded into the isolation pod by assistants wearing full. Personal Protective Equipment

**Fig. 4:**
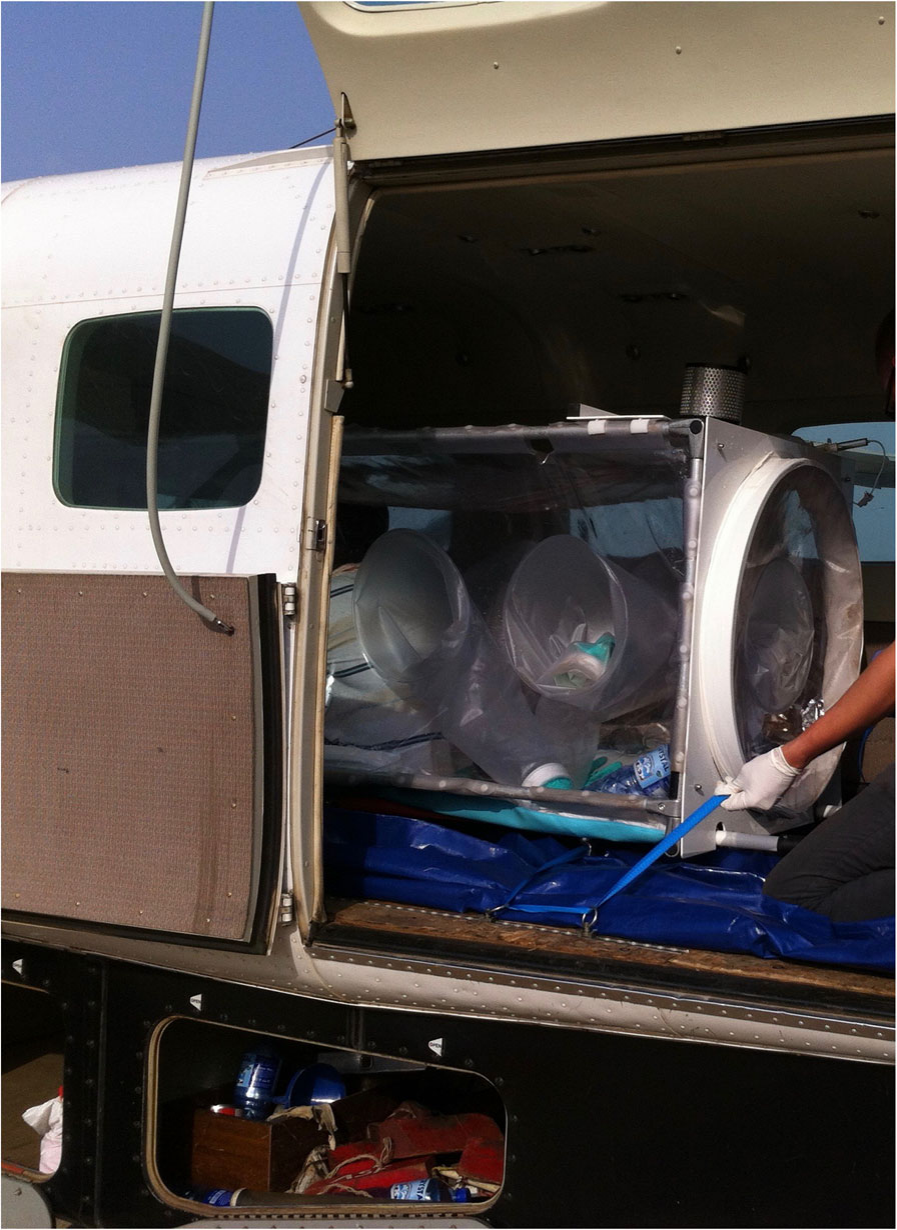
The isolation pod is attached to the floor of the aircraft

On the basis of a fever > 38.5C and a confirmed contact with an EVD case, immediate evacuation to the CTS is decided, without waiting for the results of the first RT-PCR. The “HSTI-TCOL” is prepared near the aircraft [Figs. 1 and 2]. The ambulance carrying the sick HW is parked close to the stretcher. The patient is transferred into the pod by two HWs in full PPE [Fig. 3a, b]. An IV infusion line is threaded through a dedicated aperture in the isolation pod, allowing the delivery of intravenous fluids and medication to the patient if required in flight. Before being loaded on the aircraft, and once the pod has been sealed with the patient within, the outside is sprayed with chlorine solution by staff in PPE. The air depression system is checked before departure. Four HWs ensure the stretcher installation in the aircraft. The patient is not fastened inside but the pod is strongly tied to four tie-down fittings inside the aircraft [Fig. 4]. All wastes carrying infectious risks are collected to be incinerated. All the patient’s needs during the flight are anticipated and placed inside the pod before the door is closed.

The crew is composed of a pilot and a co-pilot, a doctor and a nurse from the ETC. The flight altitude is 10500 ft (3200 m); flight duration is 2 h by airplane and 4 h with a refueling stop by helicopter, without pressurization. Full PPE is provided on the plane for all crew members, in case of unplanned pod failure. After landing in Conakry, the patient is immediately transported and supported by the CTS medical team, into the isolation area. The plane is decontaminated using a chlorine solution at every point of contact between the pod and the plane, which take about 15 min. At the end of the evacuation, when the patient’s EVD status is known, if negative the pod is cleaned with a chlorine solution; if positive the pod is incinerated, the metal frame and the engine of the pod are strictly decontaminated.

## Technical features of the HSTI—TCOL

The size is 2300 mm L x 800 mm W x 1100 H mm, and the weight is 45 kg. It includes a stretcher, 4 grips, a transparent PVC envelope with 8 gloves attached to a metallic frame, a filtration and depression engine with 2 high efficiency particulate air filters (HEPA—H14, certified efficiency of 99.997%), a manometer, a thermometer and a hygrometer. The air depression device works with an electric engine powered by two 12 V batteries and generates a depression between 2 and 5 mm of water. Batteries life is about 6 h. Ambient air penetrates through the filter attached outside the pod. Vitiated air is eliminated by a second filter screwed inside. Air renewal is 20–30 volumes per hour. A heavier version (112 kg), the Vickers Aircraft Transport Isolator (VATI), is also available but it can only be used in cargo planes and it needs 8 to 11 HWs for implementation [7, 8].

## Discussion

The first aeromedical evacuations for suspected lethal infectious diseases were performed in the 1970s for patients with Lassa fever [9]. At the same time, closed system isolators were developed to manage the risk of secondary infection of health and transport staff involved in the evacuation [7, 8, 10].

Under the evacuation protocol, once an evacuation had been agreed, ETC contacted the CTS, and together they co-ordinated with the World Food Program, to evacuate patients to Conakry. The cost of the evacuations (estimated at US$4000 per hour or about US$20,000 for the round trip from Conakry) was met from various sources including UN, EU and the French government. Due to the delays in obtaining the initial RT-PCR results in N’Zerekore, the desire to move patients before they deteriorated and became unfit for evacuation [6], and the fact that the evacuation aircraft operated only during daylight hours, the decision to evacuate all four patients was made early in their illness, based on high-risk exposure to EVD and symptoms compatible with EVD. Step by step evacuation protocols were issued to all logistic and medical staff during their briefings for work in the ETC [11]. There were 3 isolation pods available in Guinea; one in N’Zerekore, one with MSF in Conakry and one with the UN.

HSTI-TCOL small height and weight offers great advantage when used on a helicopter or a light aircraft as « CESSNA CARAVAN ». The assembly of the pod is fast and easy which allows being quickly operational. The size of this pod is also very suitable for the road transfer in a 4 wheel drive vehicle to the airport before aerial evacuation. In addition, the number of PPE dressed people needed for preparing the patient and the transfer is lower than for the heavier version of the pod, « VATI » that weighs 112 kg and double the amount of staff needed. Finally, this stretcher allows a closed monitoring of the patients.

But the pod has also a few disadvantages. First, the patient in the pod is not tied and that can be dangerous in case of in-flight turbulences. Secondly, due to the limitations of medical care possible in the cramped conditions during transport, the patient has to be stable, conscious and not agitated, without haemodynamic or respiratory disturbance. Once the pod is sealed, there is no further access without breaching infection control. Thus if a patient deteriorates in-flight, intervention was limited to IV rehydration, anti-emetics and IV sedation. Only patients well enough to survive transport should be evacuated. Internal temperature drops considerably during flight due to the altitude and lack of cabin heating [Table 1]. This highlights the need for an early decision to evacuate a patient with symptoms compatible with EVD, to minimize the risk of in-flight deterioration.

## Conclusions

The establishment in West Africa of ETCs in the struggle against the current EVD epidemic exposes health professionals to the risk of being infected by the virus. In Guinea, a treatment center dedicated exclusively to HW providing treatment to health care workers with possible EVD patients was opened by the French Government. The major ETCs in Guinea had access to HSTI-TCOL isolation pods to allow HW patients to be transported by air. The N’Zerekore ETC used the isolation pod and air transport to evacuate HW suspected of having EVD, to the CTS in Conakry. Air evacuations using the pod require that the patient does not have critical dysfunction of any major organ system which might cause deterioration in flight. Clinicians require agreed transport protocols and close co-ordination between the numerous groups involved [12, 13]. These 4 evacuations, though ultimately not involving any EVD patients, allowed the service to test its protocols and equipment, notably the HSTI-TCOL isolation pod. Thanks to effective communication and coordination between the different services, the 4 HWs suspected of having EVD were evacuated by air without breach of biosafety precautions.
